# Development of 3D-Printed Self-Healing Capsules with a Separate Membrane and Investigation of Mechanical Properties for Improving Fracture Strength

**DOI:** 10.3390/ma16165687

**Published:** 2023-08-18

**Authors:** Taeuk Lim, Hao Cheng, Jie Hu, Yeongjun Lee, Sangyou Kim, Jangheon Kim, Wonsuk Jung

**Affiliations:** 1 School of Mechanical Engineering, Chungnam National University, Daejeon 34134, Republic of Korea; 2 Department of Mechanical Engineering, Korea Advanced Institute of Science and Technology (KAIST), 373-1, Daejeon 34141, Republic of Korea

**Keywords:** 3D-printed capsule, mechanical property, separation membrane, isotropic fracture strength, compression experiment

## Abstract

Studies on self-healing capsules embedded in cement composites to heal such cracks have recently been actively researched in order to improve the dimensional stability of concrete structures. In particular, capsule studies were mainly conducted to separately inject reactive healing solutions into different capsules. However, with this method, there is an important limitation in that the probability of self-healing is greatly reduced because the two healing solutions must meet and react. Therefore, we propose three-dimensional (3D) printer-based self-healing capsules with a membrane structure that allows two healing solutions to be injected into one capsule. Among many 3D printing methods, we used the fusion deposition modeling (FDM) to design, analyze, and produce new self-healing capsules, which are widely used due to their low cost, precise manufacturing, and high-speed. However, polylactic lactic acid (PLA) extruded in the FDM has low adhesion energy between stacked layers, which causes different fracture strengths depending on the direction of the applied load and the subsequent performance degradation of the capsule. Therefore, the isotropic fracture characteristics of the newly proposed four types of separated membrane capsules were analyzed using finite element method analysis. Additionally, capsules were produced using the FDM method, and the compression test was conducted by applying force in the x, y, and z directions. The isotropic fracture strength was also analyzed using the relative standard deviation (RSD) parameter. As a result, the proposed separated membrane capsule showed that the RSD of isotropic fracture strength over all directions fell to about 18% compared to other capsules.

## 1. Introduction

Over the past few years, many researchers have developed promising techniques for the internal healing of concrete cracks using encapsulated limestone-producing bacterial spores [1,2,3,4,5,6]. This eco-friendly approach is attractive and plausible; however, bacterial self-healing is a slow low-speed approach. In addition, it is only suitable for surfaces [7,8], fractures [9,10,11], and structures located in areas with high relative humidity [12,13,14,15] or in direct contact with a significant number of microorganisms. The disadvantage of the technique is that it is difficult to apply in scenarios involving marine structures and sewage pipes. However, capsules containing self-healing fluids can use various self-healing materials to reduce their environmental impact [16]. The shells of self-healing capsules applied to concrete must have adequate breaking strength. Moreover, they should be able to rupture easily to release components under the crack-induced stress of the cement structure. However, they must be sufficiently strong to endure the mixing and curing of concrete.

Several studies have been conducted on capsule fabrication using various materials, including gelatin [17], wax [18], paraffin [19], polyurethane [20,21,22,23], glass [24,25], ceramics [26], silica [27], and silica gel [28]. However, existing capsules have been widely used in the past for self-healing encapsulation, but they have the disadvantage that fractures are common during the cement manufacturing process and require additional structures for protection. Additionally, the thickness of these shells decreases the amount of self-healing liquid, which reduces the stability of the concrete structure owing to the weakening of the mechanical strength of the concrete rather than the self-healing effect.

In addition, a major problem with existing capsules is due to the structure in which monomers and initiators used as self-healing fluids are forced to be stored individually in different capsules. These monomers and initiators are randomly dispersed in concrete, and when a crack occurs in the concrete, the two solutions may not come into contact and fail to cause a reaction. This is a major disadvantage because the two solutions may not meet if the capsule distribution is not uniform [29,30].

Therefore, in order to solve these problems, research on capsule production using 3D printing technology has recently been conducted [31,32,33]. The fabrication of capsules via 3D printing using fused deposition modeling (FDM) has very high reproducibility [31,32], uniformity [34,35], and a high degree of shape freedom [36,37]. Additionally, the 3D- printed capsule has the advantage of extending the life of the solution and capsule by separating and storing the monomer and initiator. In addition, the design of the shell thickness and structure allows for control of the fracture strength of the capsule.

However, until now, 3D-printing-based capsules had only one healing solution for each capsule. Although this has strengths compared to previous studies [17,18] in terms of fracture strength, it has the same disadvantage that monomers and initiators from different capsules must converge through capillary-action-induced cracks for the chemical reaction of self-healing. Additionally, it exhibits anisotropic mechanical strength owing to the fabrication characteristics of the FDM method; accordingly, it also has different breaking strengths depending on the direction of the load [38,39,40,41,42]. In order to solve this problem, several studies have focused on improving the mechanical strength by adjusting the filler added to the polymer matrix [43,44].

Therefore, we propose a new capsule design based on 3D printing and FDM technology that can store two self-healing solutions in one capsule in a separate state. Additionally, this study designed a capsule structure to improve the isotropic fracture strength characteristics of a two-liquid self-healing capsule with a separator. Particularly, the optimal design was established through the thickness and shape design and verification of the connection part between the separation membrane and the shell of the capsule, respectively. 

## 2. Materials and Methods

The fracture strength in each direction was analyzed using ANSYS simulations and flat plate compression experiments. The fracture strength of capsules with separation membranes in each direction was analyzed using a compression test, and it was verified that the optimal design has uniform fracture strength in all directions through RSD parameter analysis. 

In this study, a PLA material widely used in the FDM method was used based on 3D printing with excellent reproducibility, implementation, and mechanical properties [31,32]. Additionally, PLA materials have the advantage of being able to design isotropic fracture strength through optimal design and have chemical stability [43,44].

**Figure 1:**
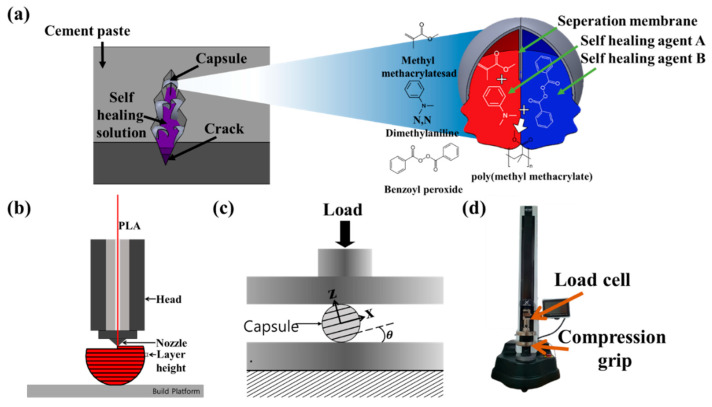
(**a**) Schematic of the 3D printing capsule-based self-healing approach; (**b**) 3D-printed capsule fabrication schematic; (**c**) schematic of the FDM-based capsule compression test and definition; (**d**) machine for compression test.

A schematic of a capsule manufactured by designing a separator structure is shown in Figure 1a. Figure A5 in Appendix A also shows the overall process of making capsules, injecting heterogeneous self-healing solutions, and applying them to cement structures. The capsule was designed such that one capsule could release a self-healing solution instantly when breaking. The capsule, having a diameter of 15 mm with a nozzle of diameter 0.4 mm, was printed at a temperature of 210 °C, layer height of 0.06 mm, and speed of 50 mm/s, with a polylactic acid (PLA) produced using the FDM method, as shown in Figure 1b [31]. The PLA (CAS Number: 26100-51-6, Molecular Weight: ~60,000) used could be present in cement for a significant time because in the distilled product, 90–99% of l-l-lactic acid could be present. The 90% pure-phase lactic acid contained impurities, such as arsenic (<1 ppm), iron (<5 ppm), heavy metals (<5 ppm), chloride (<10 ppm), sulfate (<10 ppm), sulfate (up to 0.05%), reducing sugars, methanol, and methyl ester [45]. As shown in Figure 1c, a compression test was conducted (with a compression load in the three-axis directions) to measure the mechanical strength according to the direction of the FDM capsule. Based on the PLA properties produced through the FDM method in Table A1, buckling and breaking strength due to capsule compression were analyzed. The compression test of the capsules was conducted at a speed of 10 mm/s by placing the capsules between plates with reference to the capsule strength measurement method of an existing study (using UNITEST M1 equipment from TEST ONE) [31,46,47,48,49].

## 3. Results

**Figure 2:**
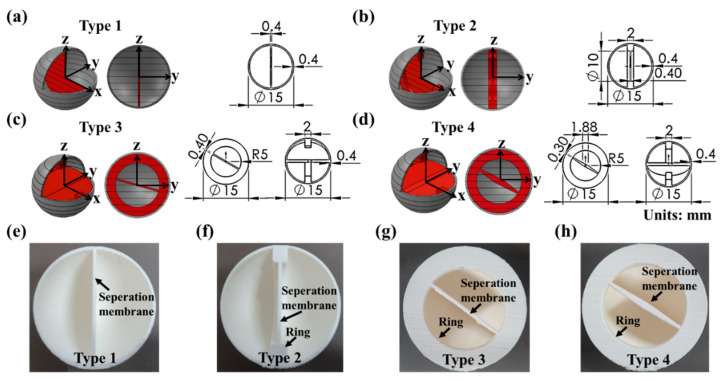
Detailed design of four types of capsules: (**a**,**e**) Type 1, (**b**,**f**) Type 2, (**c**,**g**) Type 3, (**d**,**h**) Type 4.

Figure 2a–d show capsules with four structures and values by region. Figure 2e–h show cross-sectional photographs of the capsules. Figure 2a shows the dimensions of a type 1 capsule with a structure, where a flat separator passing through the center was added to a hollow sphere-shaped capsule (in a direction perpendicular to the laminating surface). Figure 2b shows the dimensions of a type 2 capsule, where a ring-shaped support was added to the type 1 capsule parallel to the flat plate. Figure 2c shows the dimensions of a type 3 capsule obtained by adding a ring structure (perpendicular to the stacked surface) and a flat separator (forming an angle of 45° with the stacked surface) to the hollow spherical capsule. Figure 2d shows the dimensions of a type 4 capsule, where the separator cross-section of a type 3 capsule was changed to a rhombus.

### 3.1. The Finite Element Method Analysis of 3D-Printed Capsules

**Figure 3:**
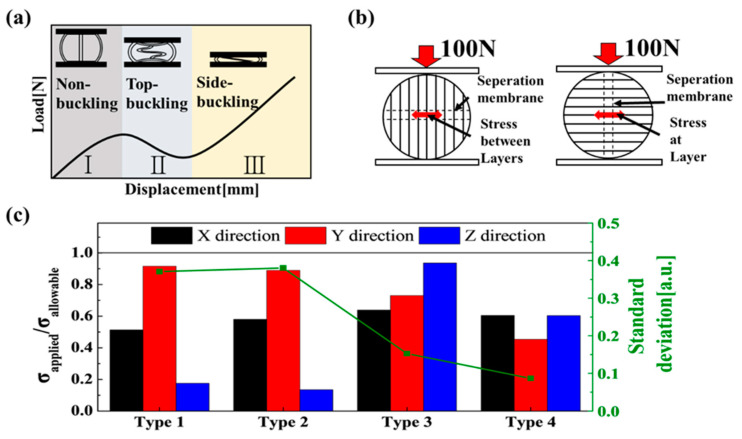
(**a**) General compression process of a sphere with a separation membrane; (**b**) major stress factors according to the applied load and printing angle; (**c**) ANSYS simulation results of the applied strength using the allowed strength according to the direction of the applied load.

Figure 3a shows the load behavior when compressing a capsule with a separator between the plates; it also shows the shape of the capsule. The capsule with separation membranes was compressed in area I, where the sphere and reputation were attached. In area II, buckling occurred in the area separator, reducing the support load. In area III, as the support load increased, sphere compression occurred. Figure 2b shows the most significant load acting on vulnerable areas along the direction of compression and the resulting fracture. For compression in the x- and y-directions, the tensile stress acts between laminations. For compression in the z-axis direction, the tensile stress tearing the middle layer was the primary load involved in the fracture. The compression of type 1–4 capsules in each axial direction was interpreted through the finite element method (FEM). The maximum tensile load applied near the equator of the ball (the vulnerable area) was divided by the allowable strength, as shown in Figure 3c. The FEM analysis was performed using the ANSYS static structure to manufacture a 15 mm diameter capsule with a compressed plate of size 20 × 20 × 10 mm (w × d × h). The structure was compressed to 100 N. The mechanical properties of PLA fabricated using the FDM method were the same as those in a study by Lim et al. (2021) [31]. For compression in the x- and y-directions, the tensile stress acted between the laminations. For compression in the z-axis direction, the tensile stress tearing the middle layer was the main load involved in the fracture. The y-axis value in Figure 3c is a comparison of the value obtained by dividing the tensile load by the allowable strength using FEM analysis. The closer the value was to 1, the higher the probability of fracture. For the type 1 capsule, the x- and y-axis compression values were 0.51 and 0.92, respectively, which were up to 5.1-times the value on the z-axis (0.18). For the type 2 capsule, the x- and y-axis compression values were 0.58 and 0.89, respectively, which were up to 6.8-times the value on the z-axis (0.13). For the type 3 capsule, the x- and y-axis compression values were 0.63 and 0.73, respectively, which were up to 1.5-times the value on the z-axis (0.93). The reinforcing structure of the type 2 capsule hardly affected the fracture. However, the inclined separator structure of the type 3 capsule reduced the difference in the predicted fracture values owing to compression in both directions. For the type 4 capsule, the x- and y-axis compression values were 0.6 and 0.45, respectively, which were up to 1.3-times the value on the z-axis (0.6). Through this, when the stacking plane and the separator are orthogonal, stress concentration occurs within the separator, leading to increased z-axis compressive strength and a further enhancement in anisotropic strength characteristics. To enhance strength isotropy, achieving a homogeneous distribution of stress applied to both the separator and capsule is of the utmost importance. For these reasons, a separator with an inclined parallelogram cross-section of a type 4 capsule was most suitable for capsule structures with isotropic strength. 

### 3.2. Fracture Strength Tests of 3D-Printed Capsules

**Figure 4:**
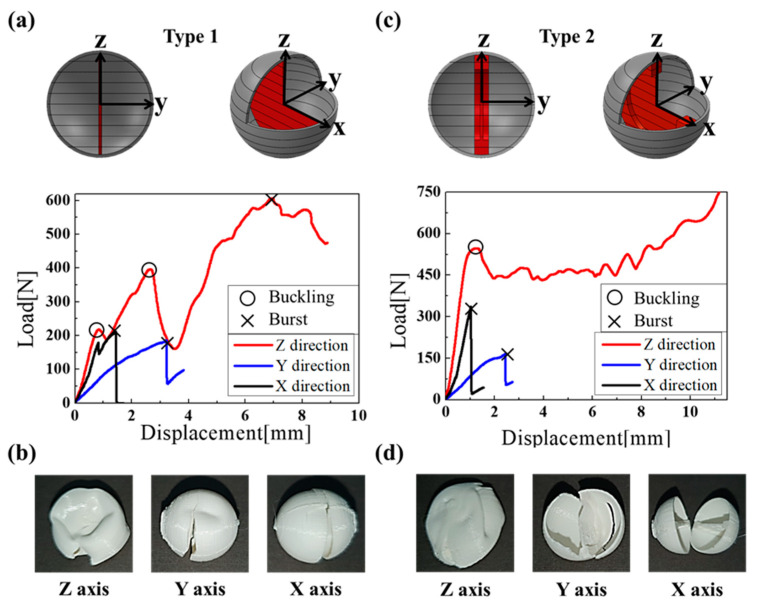
Compressive displacement according to applied load for: (**a**) type 1 and (**c**) type 2, and fractured capsule images of (**b**) type 1 and (**d**) type 2.

In Figure 4a, the type 1 capsule broke at 210.8 N when the plate mounted on the equipment was precisely 1.44 mm when compressed on the x-axis. For the y-axis, when a 3.18 mm compression was applied, the fracture occurred at a load of 182.9 N. For z-axis compression values of 0.85 and 2.61 mm, buckling occurred at loads of 217.7 and 396.7 N, respectively. For the same axis, when compressed to 6.95 mm, the fracture occurred at a load of 606.1 N. As shown in Figure 4b, the type 1 capsule broke in all three axial directions. In Figure 4c, the type 2 capsule broke at a load of 335.4 N when it was compressed to 1.03 mm along the x-axis. For the y-axis, when a 2.42 mm compression was applied, the fracture occurred at a load of 164.3 N. For the z-axis, buckling occurred at a load of 547.2 N when a 1.24 mm compression was applied. Additionally, the fracture did not cease. As shown in Figure 4d, the type 2 capsule broke only along the x- and y-axes.

**Figure 5:**
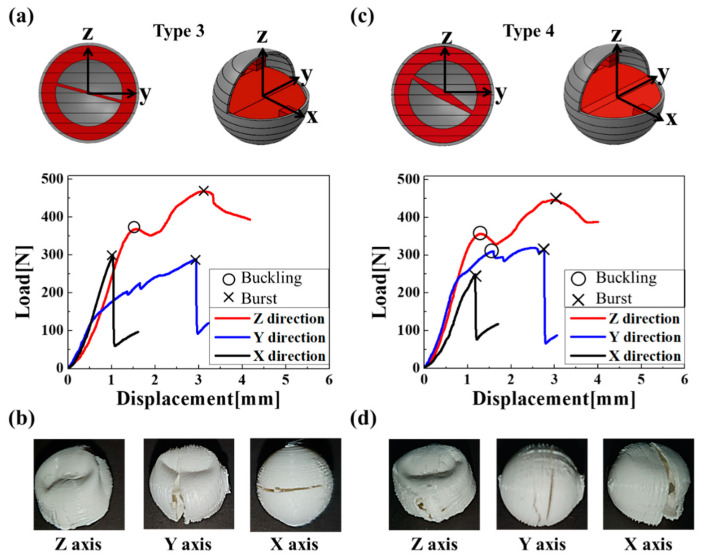
Compressive displacement according to applied load for different capsule types: (**a**) type 3 and (**c**) type 4, and fractured capsule images of (**b**) type 3 and (**d**) type 4.

In Figure 5a, the type 3 capsule broke at a load of 298.6 N when compressed by 1.03 mm along the x-axis. This occurred despite the capsule having an inclined membrane to reduce the difference between the y- and x-axis compression, bursting the load and causing a fracture along the z-axis. For the y-axis, when a 2.94 mm compression was applied, a fracture occurred at a load of 288.8 N. For the z-axis, buckling occurred at a load of 369.7 N when a 1.55 mm compression was applied. For the same system, a fracture occurred at a load of 468.3 N when a 3.12 mm compression was applied. As shown in Figure 5b, the type 3 capsule broke in all three axial directions. Figure 5c shows a type 4 capsule where the cross-section of the separator was shaped like a diamond. The reason for this was to increase the x-axis bursting load. For the x-axis, when a 1.17 mm compression was applied, the fracture occurred at a load of 249.1 N. For the y-axis, buckling occurred at a load of 310.4 N when a 1.58 mm compression was applied. Moreover, a fracture occurred at a load of 313.8 N when a 2.76 mm compression was applied. For the z-axis, buckling occurred at a load of 356.5 N when a 1.28 mm compression was applied. Moreover, a fracture occurred at a load of 446.2 N when a 3 mm compression was applied. Compared with the capsule in Figure 5a, the compression burst strength in the x-axis direction increased. As shown in Figure 5d, the type 1 capsule broke in all three axial directions.

**Figure 6:**
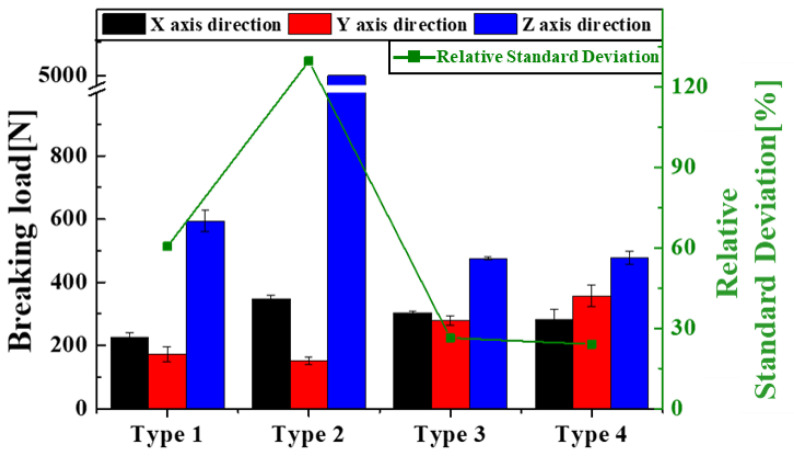
Capsule breaking load and RSD according to capsule type and direction of the applied load.

Figure 6 displays the breaking load, represented as compression resistance, along with the average and standard deviations for each capsule type compressed three times on the x-, y-, and z-axes. The type 2 capsule did not burst when compressed in the z-axis direction, so it was recorded as 5000 N, the equipment’s maximum load limit for compression tests. The type 1 capsule broke at 225.53 (±14.06), 172 (±23.38), and 593.13 N (±33.88) in the x-, y-, and z-axis compressions, respectively. The type 2 capsule broke at 346.33 (±12.66) and 151.66 N (±12.01) in the x- and y-axis compressions, respectively. No fracture occurred along the z-axis. The type 3 capsule broke at 303.33 (±6.11), 279.33 (±15.01), and 475 N (±5) in the x-, y-, and z-axis compressions, respectively. The type 4 capsule broke at 281 N (±34.17), 256.66 (±34.27), and 476.66 N (±20.81) in the x-, y-, and z-axis compressions, respectively.

## 4. Discussion

The RSD of each compression direction was analyzed to compare the intensity symmetry of the capsule. The kurtoses of the nine capsules of four types compressed in the x-, y-, and z-axes were 60.5% for type 1, 129.7% for type 2, 26.3% for type 3, and 24.1% for type 4; type 4 had the smallest RSD. This indicates that the strength of the type 4 capsule was closest to the isotropic strength, as shown in Figure 6.

**Figure A1:**
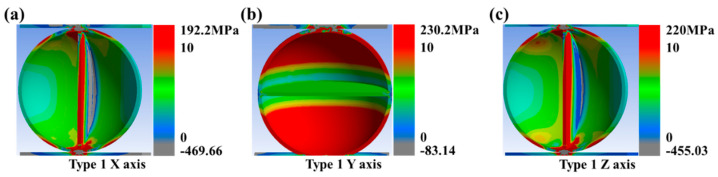
ANSYS simulation results: (**a**) type 1 x-axis compression; (**b**) type 1 y-axis compression; (**c**) type 1 z-axis compression.

**Figure A2:**
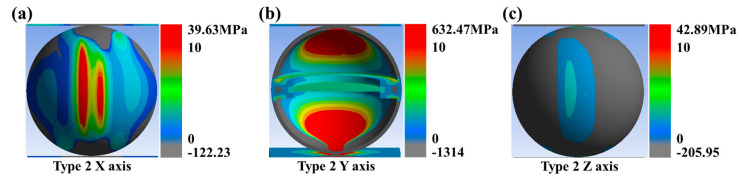
ANSYS simulation results: (**a**) type 2 x-axis direction compression; (**b**) type 2 y-axis direction compression; (**c**) type 2 z-axis direction.

In the case of type 1 and 2 capsules, when the capsule separator was perpendicular to the lamination plane, its strength was in the order of z, x, and y along the axial direction. This is because the z–x axis compression in Figure A1 and Figure A2 supported the load of the separator, which reduced the load on the vulnerable area.

**Figure A3:**
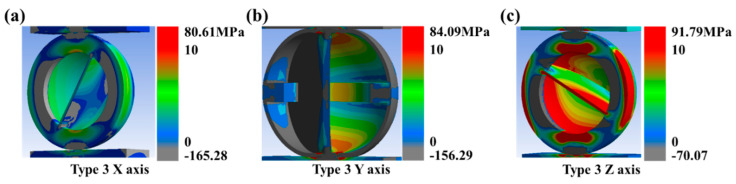
ANSYS simulation results: (**a**) type 3 x-axis compression; (**b**) type 3 y-axis compression; (**c**) type 3 z-axis compression.

**Figure A4:**
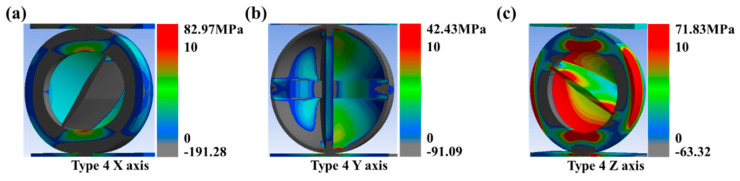
ANSYS simulation results: (**a**) type 4 x-axis compression; (**b**) type 4 y-axis compression; (**c**) type 4 z-axis compression.

However, for type 3 and 4 capsules, the separator was designed and manufactured to be inclined, not vertical. Through this design, the strength in the x-axis direction and the y-axis direction increased, and the RSD in the z-axis direction increased, resulting in a decrease in the overall deviation. As shown in Figure A3 and Figure A4, the load on the vulnerable area increased in the z-axis compression, and, on the other hand, the load on the vulnerable area decreased in the x- and y-axis compression.

Additionally, in the case of capsules with tilted separators, it was confirmed through FEM analysis and actual compression experiments that the strength isotropy was improved. Comparing Figure A3 and Figure A4, there is an insignificant change in compression in the z-axis direction. However, the vulnerable area decreased in compression in the x- and y-axis directions. A lower load on the vulnerable area indicates an increase in strength. In addition, in the case of type 3 and 4 capsules, as shown in Figure 6, the y-axis strength increased, and the RSD decreased when the cross-section of the capsules had a diamond shape. The membrane with the rhombus-shaped cross-section improved its isotropic strength characteristics by increasing its strength against compression in the y-axis direction. This was because the separator of the rhombus-shaped cross-section was larger in volume than that of the flat plate shape when the y-axis was compressed; therefore, it supported a larger load. In addition, the support load of the rhombus cross-section separator was not significantly different from that of the flat separator in x-axis compressions; thus, only the strength in the y-axis direction could be increased.

When comparing and analyzing the results of the experiment with previous studies, these previous studies have shown that most microfluidic-fabricated capsules can only contain a single solution, and containing two or more solutions results in poor shape reproducibility [44,45,46]. Furthermore, the universal method studied to date for overcoming the anisotropic properties, which is a disadvantage of FDM methods, has been to add coupling agents or filament materials to increase the adhesion energy. The 3D-printed two-liquid capsule proposed in this study structurally solves the drawbacks of lamination processing and can easily produce more complex structures than conventional capsule manufacturing methods. However, for accurate analysis and verification of the homogeneity of capsule destruction, it is believed that more accurate results can be obtained if SEM images, Keynce photos, or scratch tests are performed in future studies.

Therefore, the new type of capsule proposed in this paper is a manufacturing method that can reproduce a shape that is difficult to realize with conventional methods and maximize the isotropic performance of the capsule. In addition, it is necessary to thoroughly analyze the behavior of the self-healing solution on the fracture surface using SEM for mixing and dispersion of the self-healing solution due to fracture.

## 5. Conclusions

**Figure A5:**
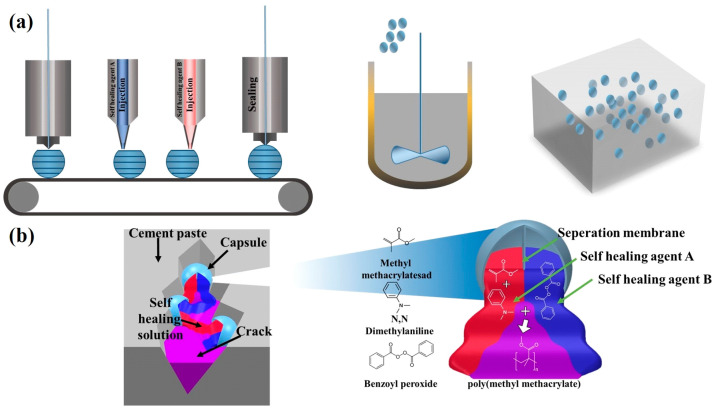
(**a**) Fabrication of two-component self-healing capsules and application process to concrete. (**b**) Schematic of solution dispersion for crack healing of two-component self-healing capsules.

In summary, this study proposed a novel two-component self-healing capsule structure that overcame the disadvantage of the anisotropic strength of 3D-printing-based fabrications using FDM methods. An ANSYS FEM analysis was performed to design capsules with isotropic fracture strengths. Based on the analysis results, two liquid capsules with four structures were manufactured using FDM 3D printing, and compression experiments were conducted. Figure 6 shows that the type 3 and 4 capsules had lower *z*-axis strength and higher x- and *y*-axis strength than those of type 1 and 2 capsules. In addition, the type 4 capsule showed the lowest standard deviation of 86.41 MPa. Moreover, the strength in the *x*-axis direction was stronger than that in the *y*-axis direction when compared to that of the type 3 capsule. This affected the strength in a specific direction depending on the slope of the separator of the capsule and the shape of the cross-section. Accordingly, it was possible to induce isotropic strength in the capsule manufactured using the FDM method. This was realized by adjusting the tilting angle and cross-sectional shape of the separator. As shown in Figure A5, capsules with improved strength and isotropic properties do not require a special alignment process in the concrete mixing process, and even if arranged arbitrarily, they are broken by the same breaking stress, regardless of direction, releasing a self-healing solution.

Structural and analytical studies of the proposed capsules will be useful in improving the isotropic fracture strength and accelerating 3D-print-based self-healing capsule studies. Additionally, in order to apply self-healing capsules to building structures in the future, research is needed on injecting heterogeneous self-healing solutions into manufacturing capsules and establishing a mass production system based on the sealing process. In addition, when applied to concrete, there is a need to analyze the adhesion strength between the capsule and concrete to ensure simultaneous rupture with the crack in the future.

## Data Availability

Not applicable.

## Appendix A

**Table A1:** Mechanical properties of PLA specimens manufactured using the FDM method.

Mechanical Properties	Values
Tensile strength [*σ*_X,Y_]	50.62 [MPa]
Tensile strength [*σ*_Z_]	8.46 [MPa]
Shear strength [τ_XY_]	13.53 [MPa]
Shear strength [τ_YZ,XZ_]	29.56 [MPa]
Compression strength [*σ*_X,Y_]	−26.44 [MPa]
Compression strength [*σ*_Z_]	−50.35 [MPa]
Young’s modulus [E_X,Y_]	3.14 [GPa]
Young’s modulus [E_Z_]	2.19 [GPa]
Shear modulus [G_XY_]	0.981 [GPa]
Shear modulus [G_YZ,XZ_]	0.937 [GPa]
Poisson ratio [ν_XY_]	0.28 [a.u.]
Poisson ratio [ν_YZ,XZ_]	0.33 [a.u.]
